# A New Intra-Specific and High-Resolution Genetic Map of Eggplant Based on a RIL Population, and Location of QTLs Related to Plant Anthocyanin Pigmentation and Seed Vigour

**DOI:** 10.3390/genes11070745

**Published:** 2020-07-04

**Authors:** Laura Toppino, Lorenzo Barchi, Francesco Mercati, Nazzareno Acciarri, Domenico Perrone, Matteo Martina, Stefano Gattolin, Tea Sala, Stefano Fadda, Antonio Mauceri, Tommaso Ciriaci, Francesco Carimi, Ezio Portis, Francesco Sunseri, Sergio Lanteri, Giuseppe Leonardo Rotino

**Affiliations:** 1 CREA, Research Centre for Genomics and Bioinformatics, 26836 Montanaso Lombardo (LO), Italy; laura.toppino@crea.gov.it (L.T.); tea.sala@crea.gov.it (T.S.); stefano.fadda@crea.gov.it (S.F.); giuseppeleonardo.rotino@crea.gov.it (G.L.R.); 2 DISAFA–Plant genetics and breeding—University of Turin, 10095 Grugliasco (TO), Italy; matteo.martina@unito.it (M.M.); ezio.portis@unito.it (E.P.); sergio.lanteri@unito.it (S.L.); 3 Institute of Biosciences and BioResources, Division of Palermo—National Research Council (CNR), 90129 Palermo, Italy; francesco.mercati@ibbr.cnr.it (F.M.); francesco.carimi@ibbr.cnr.it (F.C.); 4 CREA, Research Centre for Vegetable and Ornamental Crops, 63030 Monsampolo del Tronto (AP), Italy; acciarri@libero.it (N.A.); t.ciriaci@libero.it (T.C.); 5 CREA, Research Centre for Vegetable and Ornamental Crops, 84098 Pontecagnano (SA), Italy; domenico.perrone@crea.gov.it; 6 CREA, Research Centre for Plant Protection and Certification, 84091 Battipaglia, Italy; 7 Institute of Agricultural Biology and Biotechnology (IBBA)—National Research Council (CNR), 20133 Milano, Italy; stefano.gattolin@ibba.cnr.it; 8 Department AGRARIA, University Mediterranea of Reggio Calabria, 89124 Reggio Calabria, Italy; antonio.mauceri87@unirc.it (A.M.); francesco.sunseri@unirc.it (F.S.)

**Keywords:** linkage map, RAD, QTL, *Solanum melongena*

## Abstract

Eggplant is the second most important solanaceous berry-producing crop after tomato. Despite mapping studies based on bi-parental progenies and GWAS approaches having been performed, an eggplant intraspecific high-resolution map is still lacking. We developed a RIL population from the intraspecific cross ‘305E40’, (androgenetic introgressed line carrying the locus *Rfo-Sa1* conferring *Fusarium* resistance) x ‘67/3’ (breeding line whose genome sequence was recently released). One hundred and sixty-three RILs were genotyped by a genotype-by-sequencing (GBS) approach, which allowed us to identify 10,361 polymorphic sites. Overall, 267 Gb of sequencing data were generated and ~773 M Illumina paired end (PE) reads were mapped against the reference sequence. A new linkage map was developed, including 7249 SNPs assigned to the 12 chromosomes and spanning 2169.23 cM, with iaci@liberoan average distance of 0.4 cM between adjacent markers. This was used to elucidate the genetic bases of seven traits related to anthocyanin content in different organs recorded in three locations as well as seed vigor. Overall, from 7 to 17 QTLs (at least one major QTL) were identified for each trait. These results demonstrate that our newly developed map supplies valuable information for QTL fine mapping, candidate gene identification, and the development of molecular markers for marker assisted selection (MAS) of favorable alleles.

## 1. Introduction

Eggplant (*Solanum melongena* L., 2n = 2x = 24) is a member of the Solanaceae, a large plant family comprising over 3000 species and including important crops such as tomato, potato, pepper and tobacco. Unlike most of the other solanaceous crops, which are native to the New World [1,2,3], eggplant has a phylogenetic uniqueness due to its exclusive Asian origin [4]. It has been reported that the species resulted from two or three independent domestication events [5,6], although a recent study suggested a single domestication event [7]. Eggplant worldwide production is estimated as about 54 Mt, with China, India and Indonesia being the major producing countries, while Egypt, Turkey and Italy represent the main producers in the Mediterranean region (FAO 2018; [8]). Breeding efforts in eggplant, like in most crops, have been focused on increasing yield, resistance/tolerance to biotic and abiotic stress, and fruit shelf-life, but also on improving some plant morphological distinguishing traits (reduced prickliness and leaf hairiness) as well as raising the content of health-promoting metabolites (e.g., anthocyanins and chlorogenic acid) or reducing the anti-nutritional content (e.g., steroidal glycoalkaloid, saponins) in the berries. Furthermore, studies have been carried out with the goal to improve seed germination and seedling emergence [9,10,11], which affect the crop performance. 

In eggplant, several inter-specific genetic maps were developed by applying pre-next generation sequencing (NGS) techniques (RFLP, AFLP, RAPD, SSR, etc.). They were based on inter-specific crosses between cultivated *S. melongena* and *S. linnaeanum* (=*S. sodomaeum)* or *S. incanum* and used for Quantitative Trait Loci (QTL) analyses of domestication and morphological traits [12,13,14,15,16], as well as to locate genes involved in polyphenol biosynthesis [17] and resistance to *Verticillium* spp. [18]. Intra-specific maps were also built [19,20,21,22] and, more recently, Fukuoka et al. [23] generated two intra-specific genetic maps based on F_2_ populations, which were then combined into one on the basis of common markers for studying macro-syntenic relationships between eggplant and tomato, as well as for QTL analysis of parthenocarpy [24] and resistance to *Fusarium oxysporum* [25]. 

The advent of NGS-based marker technologies, by increasing the speed, throughput, and cost effectiveness of genotyping and providing genome-wide marker coverage, has allowed the development of the so-called ‘second generation’ maps. Barchi et al. [26], by applying the RAD-seq protocol from Baird et al. [27] on an intra-specific F_2_ population, identified 10,000 single nucleotide polymorphisms (SNPs) as well as nearly 1000 polymorphic indels, and more than 2000 SNPs were found of potential use for genotyping on the basis of a GoldenGate© assay. Afterwards, the first ‘second generation genetic map’ was developed [28], which included 415 markers assigned to the 12 chromosomes. The latter was used to identify the genetic bases of traits associated with anthocyanin content [28] and, more recently, for detecting QTL affecting key horticultural traits [29], fruit metabolic content [30] and resistance to soil-borne diseases [31]. Furthermore, the previously identified loci were validated, and new linked marker/trait associations were detected, through a genome-wide association (GWA) mapping approach [32,33]. Second generation intra-specific genetic maps were also generated [2] for anchoring the first draft genome sequence of eggplant and for mapping resistance QTLs to *Ralstonia* strains by SNPs developed through Illumina sequencing of the parents of a Recombinant inbreed line (RIL) mapping population as well as AFLP, SSR and SRAP markers [34].

Despite recent efforts, the linkage maps used for identifying the genetic basis of traits of breeding interest are still not saturated, hampering the fine mapping of QTL regions and the identification of candidate genes associated with the phenotypic traits. Up to now, the only available high-resolution SNP-based linkage map was developed on a F_2_ population from the inter-specific cross (*S. melongena* × *S. linneanum*) and was employed to highlight QTLs affecting stem height and fruit and leaf morphology [35]. 

We previously developed a RIL mapping population of 170 F_6_-F_7_ lines from the intra-specific cross between the breeding lines ‘305E40’ (female parent) and ‘67/3’ (male parent). Furthermore, the first high quality eggplant genome sequence of the breeding line ‘67/3’ was released [36] and, through the resequencing of the female parent (‘305E40’) and a low coverage Illumina sequencing of each RIL, we constructed a first linkage map aimed at anchoring the scaffolds to the 12 chromosomes. The map also demonstrated efficient mapping metabolomic traits of interest related to the metabolomics composition of fruit flesh and peel [37]. 

The genetic basis of anthocyanin synthesis and accumulation has been widely studied in the Solanaceae [38,39,40,41,42,43]. In the last decade, QTL-related studies using family-based or GWA mapping approaches allowed us to shed light on the genetic bases of anthocyanin distribution in eggplant as well as to identify its syntenic relationships with tomato [28,30,32,44]. By contrast, no information is available on QTLs controlling seed vigor in term of speed of seedling emergence, which diversifies the parents of our RIL mapping population. Here, we propose a more breeder-friendly map developed through a genotype-by-sequencing (GBS) approach with our RIL mapping population, whose reliability for mapping studies has been proved by identifying QTLs related to plant anthocyanin pigmentation and seed vigor.

## 2. Materials and Methods 

### 2.1. Plant Material

A population of 163 F_7_ plants, previously obtained by the single seed descent approach from a cross between eggplant lines ‘305E40’ and ‘67/3’ [28,36], was employed. The two parental lines were contrasting for a wide number of key agronomic and metabolic traits [28,29,30,31], as well as for their seed vigor. The ‘305E40’ line (female parent) is a double haploid derived from the inter-specific somatic hybrid [*Solanum aethiopicum* gr. gilo(+)*S. melongena* cv. Dourga], which was repeatedly backcrossed with the recurrent eggplant genotypes (lines DR2 and Tal1/1) prior to selfing and anther culture. This line carries the locus *Rfo-sa1* from *S. aethiopicum*, which confers complete resistance to the soil-borne fungus *Fusarium oxysporum f. sp. melongenae* (*Fom*) [45] and is partially resistant to *Verticillium dahliae* [31]. Plants of ‘305E40’ display a slight anthocyanin overall pigmentation, produces pink flowers and long, highly pigmented dark purple fruits characterized by the presence of the anthocyanin delphinidin-3-rutinoside (D3R) as well as a higher glycoalkaloids and organic acid content than the ones of ‘67/3’ [28,29,30]. The ‘67/3’ line is an F8 selection from the intra-specific cross cv. ‘Purpura’ × cv. ‘CIN2’, which lacks the *Rfo-sa1* locus and is fully susceptible to *Verticillium* [31]. Its plants display higher anthocyanin pigmentation than ‘305E40’ in leaves and stems and produce violet flowers and round, violet colored fruits with white peel colour both under and next to the calyx. The fruits are characterized by the presence of the anthocyanin nasunin in the peel, higher soluble solids, sugars and chlorogenic acid content in the flesh compared to 305E40. 

The mapping population was sown, along with both parents and the F_1_ hybrid, in glasshouses at Montanaso Lombardo in 2012. The seeds were sown in plastic trays consisting of 104 holes (8 rows of 13 holes each) filled with peat and placed over an electric warmed carpet at 24 °C. For each RIL, we sowed 52 seeds split in two replicates of 26 seeds. Each replicate was sown in two adjacent randomly chosen rows containing 13 holes of the replicate-specific tray, using a single seed per hole; each replicate was kept under the same conditions but in a different glasshouse at Montanaso Lombardo. All plantlets were grown in heated glasshouses (minimum temperature of 15 °C ensured) until the 3rd–4th leaf, and then were transplanted in three field trials in northern (Montanaso Lombardo, ML, 45°20′ N, 9°26′ E), central (Monsampolo del Tronto, MT, 42°53′ N,13°47′ E) and southern (Battipaglia, BP, 40°36′ N, 14°59′ E) Italy. Mulched twin rows of 1.1 m width were arranged using plastic black PE (0.05 mm), and plantlets were transplanted at 45 cm between each other along the rows. A drip irrigation system was employed for watering and fertilizing, and local standard horticultural practices were applied. In each site, the material was transplanted in the field according to a randomized block design (3 replicate blocks, 4 plants per block) to score the phenotypic traits. 

### 2.2. Library Construction and Sequencing

DNA from the RIL population, parental lines and F_1_ hybrid was extracted following a modified CTAB method [46] as indicated elsewhere [47]. Library construction was performed as proposed by Acquadro et al. [48] by using a HindIII-MseI enzyme combination and adding a final biotin/streptavidin-coated beads-based purification step. Quality, quantity and reproducibility of libraries were assessed with a Bioanalyzer instrument (DNA High Sensitivity chip) as well as qPCR using KAPA SYBR FAST Universal 2X qPCR Master Mix (Kapa Biosystems, Boston, MA, USA). On the basis of the quantitation, DNA libraries were pooled and sequenced on Illumina HiSeq 2500 platform (Illumina Inc., San Diego, CA, USA), following the manufacturer protocol using 150 PE chemistry at Biodiversa srl (Rovereto (TN), Italy).

### 2.3. Sequence Analysis and Map Construction

Raw reads were analyzed with Scythe (https://github.com/vsbuffalo/scythe) for filtering out contaminant substrings, and Sickle (https://github.com/najoshi/sickle), for removing reads with poor quality ends (Q < 30). Illumina reads were de-multiplexed on the basis of the Illumina TruSeq index using Stacks process rad tags. Alignment to the reference eggplant genome [36] was carried out using the Burrows-Wheeler Aligner (BWA) aligner [49] (i.e., mem command) with default parameters and avoiding multiple-mapping reads. BAM files were processed and use for the SNP calling using bcftools mpileup/call/norm utilities [50] with default parameters, except for the use of multiallelic calling model (-m option), minimum mapping quality (Q = 20) and filtering out multimapping events (−q > 1). Only SNPs with at most 20% of missing data and a mean-minDP of 20 were retained for linkage analyses. Polymorphic markers were grouped in linkage groups with “R/qtl” package [51], with minimum LOD = 8, rec ≤ 0.15. For each linkage group identified, identical loci were removed with the Exclude identical function and the remaining loci were ordered with Joinmap software (version 4, [52]), using a LOD of 8 and the Kosambi function to estimate distance and the Maximum-likelihood function to infer correct order. Markers exhibiting segregation distortion were identified applying the chi-square (X^2^)-goodness-of-fit test (*p* < 0.001) and also integrated into the map. The ordering step was iterated several times, each time by correcting genotype calls with the “SMOOTH.pl” script, which is a Perl implementation of the SMOOTH software, as developed by van Os et al. [53]. Finally, visual inspection of genotypes was applied to identify and correct the remaining genotype errors. Linkage groups were visualized in MapChart (version 2.32, [54]).

### 2.4. Phenotypic Traits Evaluation

The speed of emergence and hypocotyl anthocyanin distribution traits were assessed on 56 plantlets of each RIL, of the two parental lines and their F_1_ hybrids, which were obtained from as many seeds sown as previously described in Plant material. 

**Figure 1:**
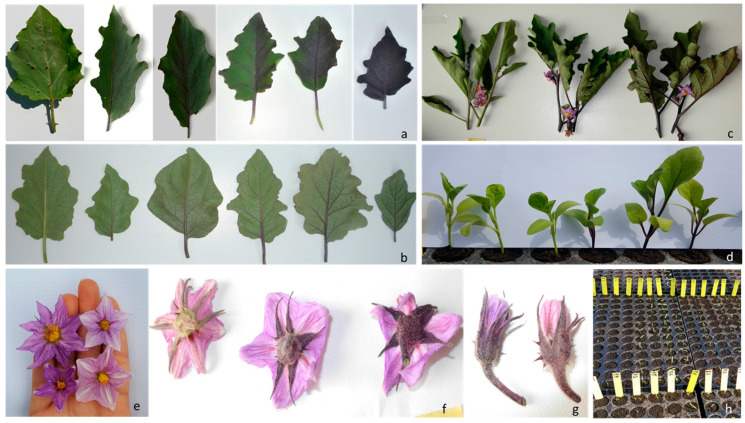
Phenotypic trait evaluation. (**a**) *adlan* (range from 0 to 5); (**b**) *lvean* (0–5); (**c**) *stean* (1,3,5); (**d**) *hyan* (0, 5); (**e**) *corcol* (clockwise from top-right: light violet, light pink, dark pink, dark violet); (**f**) *flian* (1,3,5); (**g**) *toan* (violet-reddish); (**h**) *sei* (picture at 10 days after sowing).

The speed of emergence index (*sei*) was evaluated as the time, expressed in days, needed for the emergence of 50% + 1 plantlets from the soil. Hypocotyl anthocyanin (*hyan*) trait was assessed on plantlets at the second–third leaf stage (Figure 1d) according to a 0–5 scale, with “0” representing no visible anthocyanin coloration (i.e., completely green tissues) and “5” representing complete dark violet coloration. 

In all the three field trials (ML, MT and BT), the material was arranged as a set of three randomized complete blocks with 4 replicate plants per entry per block. Phenotyping was based on the European Cooperative program for Plant Genetic Resource descriptors panel for Solanaceae (ECPGR, 2008) and the International Board for Plant Genetic Resource descriptors for eggplant (IBPGR, 1990). The traits assayed, reported in Figure 1 and detailed in Table 1, were: adaxial leaf lamina anthocyanin (*adlan*), corolla colour (*corcol*), flower anthocyanin intensity (*flian*), hypocotyl anthocyanin (*hyan*), leaf venation anthocyanin (*lvean*), stem anthocyanin (*stean*), anthocyanin tonality (*toan*). 

The anthocyanin content of stems, leaves and flower calyxes was scored on a 0 (no visible coloration) to 5 (complete dark violet coloration) scale. Anthocyanin content in leaves and leaf venation was evaluated on 4 leaves per RIL in each block, chosen in the upper middle part of the plant. Stem anthocyanin content was measured as an average value based on 3 stems per block. Flower anthocyanin intensity resulted from averaging 5 flowers per block. Anthocyanin tonality was scored as “1” reddish, “3” intermediate, or “5” violet. Finally, for the corolla colour, the trait was coded as “1”, pink; “2”, dark pink; “3”, light violet; “4”, violet-pink and “5”, violet. 

### 2.5. Statistical Analyses and QTL Detection

Statistical analyses were performed using R software [55]. A conventional analysis of variance was applied to estimate genotype and environment effects based on the linear model Y_ij_ = μ + g_i_ + b_j_ + e_ij_, where μ, g, b and e represent, respectively, the overall mean, the genotypic effect, the block effect and the error. Broad-sense heritability values were given by σ^2^_G_/([σ^2^_G_ + σ^2^_E_]/n), where “σ2G” represents the genetic variance, “σ2E” the residual variance and “n” the number of blocks. Correlations between traits were estimated using the Spearman coefficient, and normality, kurtosis and skewness were assessed with the Shapiro–Wilks test (α = 0.05). Segregation was considered as transgressive when at least one individual RIL recorded a trait value higher or lower by at least two standard deviations than the higher or lower scoring parental line. QTL detection was performed considering each location independently and was based on the newly developed map using MQM [56,57] mapping, as implemented in MapQTL v4 software [58]. QTLs were initially identified using interval mapping, after which one linked marker per putative QTL was treated as a co-factor in the approximate multiple QTL model. Co-factor selection and MQM analysis were repeated until no new QTL could be identified. LOD thresholds for declaring a QTL to be significant at the 5% genome-wide probability level were established empirically by applying 1000 permutations per trait [59]. Additive and dominance genetic effects, as well as the percentage of the phenotypic variation (PVE) explained by each QTL, were obtained from the final multiple QTL model. Individual QTLs were prefixed by a trait abbreviation, followed by the relevant chromosome designation—BT, ML or MT—which was added as a suffix when a QTL was expressed in a site-specific manner. Confidence interval of the QTL was calculated at a LODmax−1 interval or at least by considering 0.5Mb upstream and downstream (if not explicitly reported in the text) the marker identified at the QTL. CMplot was used for drawing QTL results [60]. No site-specific suffix was added to the *hyan* and *sei* QTLs, as these two traits were assessed in a single environment. 

## 3. Results

### 3.1. Sequencing and Linkage Map Construction

A total of 855 million paired-ends (PE) reads were produced, corresponding to about 257 Gb of data. After demultiplexing, cleaning and trimming, a total of 745 M Illumina PE reads were retained, corresponding to an average number of PE reads per sample of 4.48 M, with a standard deviation of 2.87 M (Figure S1). The sequence data were deposited into NCBI Short Read Archive under the Bioproject PRJNA635547.

**Figure 2:**
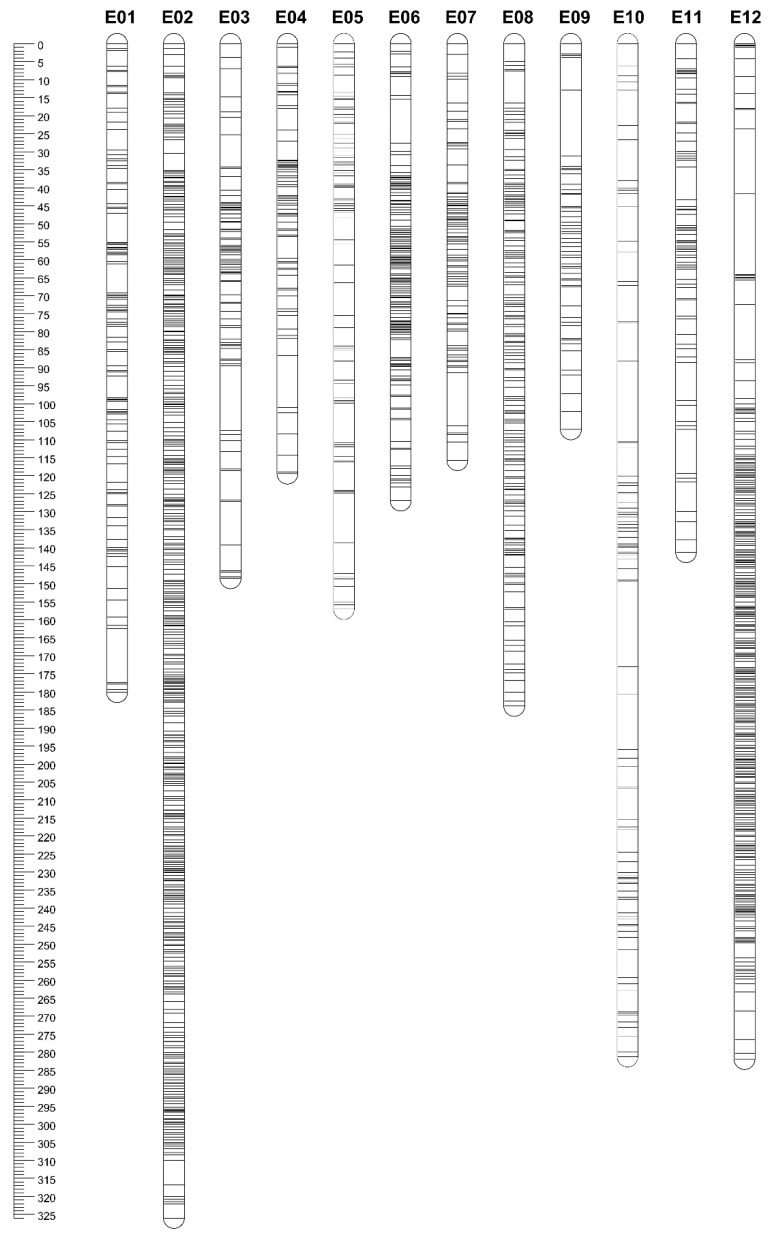
Eggplant linkage map depicting the size of the chromosomes and markers distribution. Marker names and map distances (in cM) are detailed in Figure S2.

Reads were then aligned to the reference eggplant genome [36]; close to 100% of reads were successfully mapped to single regions (no multiple mapping was permitted). A total of 10,316 polymorphic sites (i.e., markers) were identified after SNP calling using conservative filtering parameters, and were used for mapping purposes by applying a combination of R/qtl and Joinmap. At first, all markers were fed to R/qtl for linkage group identification and eventually ordered with Joinmap. Overall, 7249 markers were successfully retained and assigned to the 12 linkage groups (LG) corresponding to the haploid chromosome number of the species (Figure 2 and Figure S2). A total of 1744 markers showed segregation distortion with *p* < 0.001, covering about 24% of the total mapped markers. Chromosome E02 showed the largest segregation distortion for 1423 markers, followed by E05, with 72 markers (Table 2). 

The marker names, chromosome, and genetic position of all markers on the map and RILs haplotypes are included in Table S1. The linkage map spans 2169.23 cM (Table 2), with E02 being the longest (326 cM) chromosome and containing the highest number of markers (i.e., 1454), while E09 was the shortest (107 cM) and contained 230 markers. Some markers belonging to contigs previously assigned to CH0 [36] were mapped to the 12 LGs, of which the majority were mapped on E06, while just 47 were mapped to other chromosomes. The genome-wide mean inter-locus separation was reduced (mean of 0.4 cM), with the highest value (0.7 cM) for E05 and E10. The mapping of the SNP markers on the eggplant genome sequence revealed a total coverage of 95.88% (1095.72 Mb) of the diploid genome (1142.80 Mb). The genome-wide mean inter-locus separation was 216.5 kb, with the highest value (411.2 kb) in E04. The largest gap was observed on E12 (22.45 cM), while almost all gaps were less than 5 cM. The recombination rate of different chromosomes was estimated as the quotient between the genetic distance (cM) covered by the corresponding LG and the size in Mb of the chromosome fragment covered with markers. This value ranged from 0.81 cM/Mb on E07 to 3.91 cM/Mb on E02.

To evaluate the quality of the map obtained, we used heat maps of recombinant frequency, which highlighted that the mapped markers were ordered correctly, as the pair-wise recombination rates were noticeably low between adjacent markers (diagonal distribution of the yellow color indicates the lowest recombination rate) in the heat map for each chromosome, except E02, E06, E08 and E12 (Figure S3). Similar results were obtained when collinearity between the genetic distances of mapped SNP on each linkage group and their corresponding physical position on the eggplant chromosomes was spotted (Figure S4).

### 3.2. Phenotypic Variation and Inter-Trait Correlations

A summary of the phenotypic performance for each trait in the parental lines, hybrid F_1_ and RILs, together with the skewness, kurtosis, broad sense heritability (*h_2BS_*) values and presence of transgressive genotypes for each trait, are listed in Table 2. As expected, the parental lines contrasted for each trait. The female line ‘305E40’ had a delayed emergence from the soil compared to ‘67/3’, as evidenced by its higher *sei* (6 days). The ‘305E40’ line showed lower anthocyanin content in leaves, leaf venations, flower calyxes and stems; its hypocotyl was characterized by a reddish tonality and produced flowers with a pink corolla. Conversely, line ‘67/3’ produced violet flowers. The F_1_ hybrid’s phenotype was intermediate between the two parents for *adlan*, *toan* and *hyan*. For the remaining traits, the F_1_ hybrid was more similar to ‘67/3’ in all environments. Transgressive genotypes among the RILs were limited and only toward ‘305E40’—more precisely, three RILs for *steanBT,* two RILs for *lveanML* and *adlanBT*) and one RIL for *lveanBT*, and *hyan*. An exception was the speed of plant emergence, which was delayed in 44 RILs with respect to the late female parent ‘305E40’. The *h^2^* was overall high, ranging from 0.86 (*lveanBT*) to 0.98 (*sei* and *hyan*) (Table 2). 

Significant inter-trait correlations were detected within and across locations (Table 3), and the same traits appeared to be highly correlated in the three locations. No significant correlation was detected for *sei* with other traits as well as between *adlanMT* and traits such as *corcolBT*, *corcolML* or *hyan*. The least correlated traits were *adlan* with *hyan*, *toan* and *corcol* (in all environments), while the most highly correlated were *corcol* and *toan* in both the BT (+0.92), and ML (+0.91) environments. 

### 3.3. QTL Analysis

**Figure 3:**
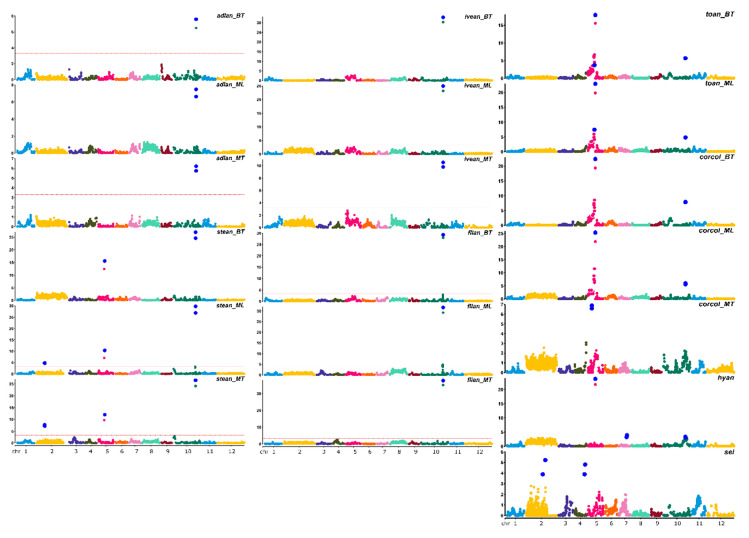
QTLs identified for the traits in study. Blue dots represent markers within the confidence interval of the QTL (LODmax−1 interval), with LOD values plotted against genome locations. Red lines in the Manhattan plots indicate LOD significance threshold.

LOD score, percentage of variance explained (PVE), and confidence interval (CI) related to QTLs, are described in Table 4. QTL analyses on all traits and environments yielded a total of 23 major (PVE values >10%) and 11 minor QTLs (Figure 3). 

QTL analyses for both *hyan* and *sei,* which were evaluated in a single location, highlighted one major and two minor QTLs. Separate analyses performed on each location for *adlan*, *lvean*, *stean*, *corcol* and *toan* resulted in the identification of a ratio between major and minor QTLs of 8/2, 7/4 and 6/1 in BT, ML and MT, respectively. The majority of QTLs identified can be considered stable, as they had the same genomic position across the three locations, with the exception of the minor QTLs *stean2.1*, confirmed both in MT and ML, but not in BT, and *corcol10.1*, demonstrating a major QTL in BT and a minor QTL in ML, and not detected in MT. Moreover, the major QTL *corcol5.1*, detected both in BT and ML, was mapped in a different position of E05 and was found as playing a minor effect in MT. For the anthocyanin-related QTLs, the positive alleles responsible for an increase in the anthocyanin content and for the presence of a violet vs. reddish pigmentation derived from ‘67/3’. Concerning the speed of emergence index, for all the QTLs but *sei2.2*, the allele increasing seed vigor derived from ‘67/3’. The largest single QTL effect was associated with *flian10.1_MT* (69.3% of the PVE). The additive effects of all the QTLs were significant at *p* < 0.05. 

All the identified QTLs were distributed over five chromosomes (Table 4), namely E02, E04, E05, E07, E10, and two evident clusters of QTLs were detected (Figure 3). One is located on E05, which also harbors two adjacent sub-clusters of QTLs conserved in the three locations. The former is comprised between 62.45 to 66.5 cM, and contained QTLs for *stean* and *toan*; the other is at 75.48 cM, and included coincident QTLs for *hyan, toan* and *corcol*. The second main cluster is on E10 at 231.77–236.98 cM and included the major QTLs *stean10.1*, *lvean10.1, adlan10.1* and *flian10.1*, and the minor QTLs *hyan10.1, corcol10.1,* and *toan10.1.*

#### 3.3.1. QTL Affecting Plant Pigmentation in Eggplant

##### Adaxial Leaf Lamina Anthocyanin (*adlan*)

A single major QTL, *adlan10.1,* was mapped on E10 at 236.987 cM next to the marker CH10_95003635 in all the three environments, and explains 21% of PVE in BT, 20.4% in ML and 17.7% in MT.

##### Stem Anthocyanins (*stean*)

Two major QTLs on E05 and E10 were detected in all the environments. The QTL *stean10.1* on E10 explains from 40% to 48% of the PVE and is located at 231.46 cM (proximal marker: CH10_94779014). The second major QTL *stean5.1*, explains from 13% to 21% of the PVE and maps on E05, within the same confidence interval (62.45–66.51 cM) in all locations (proximal marker: CH05_36124744). A third minor QTL was spotted in ML and MT, but not in BT, on E02 at 88.73 cM (proximal marker: CH02_30555633), and explains 6% and 9% of PVE, respectively. 

##### Leaf Venation Anthocyanins (*lvean*) 

One major QTL determining anthocyanin pigmentation in leaf venation (*lvean10.1*), explaining from 29% to 64% of PVE, was mapped on E10 in all locations at 231.46 cM (proximal marker: CH10_94779014).

##### Flower Anthocyanin Intensity (*flian*)

The unique major QTL *flian10.1* mapped, in all environments, on E10 at 231.46 cM (proximal marker: CH10_94779014), explains from 59% to 69% of the PVE.

##### Anthocyanin Tonality (*toan*)

Data for *toan* were only available for BT and ML environments. A major QTL (*toan5.1)* explaining 36% and 47% in BT and ML, respectively, was spotted on E05 at 75.48 (proximal marker: CH05_37533757). A minor QTL (*toan5.2*) was identified on E05 within the same CI in both locations at 61.55–66.39 cM. A third QTL with a minor effect (PVE explained from 6.8% to 9.3%) was spotted on E10 at 231.46 cM.

##### Hypocotyl Anthocyanins (*hyan*)

The QTL analysis for this trait was performed with data from one environment. A major and two minor QTLs were spotted on E05, E07 and E10, respectively. The largest effect locus (*hyan5.1*) explains 48% of PVE and is located on E05 at 75.48 cM (proximal marker: CH05_37533757). A minor QTL (*hyan7.1*), explaining 6% of the PVE, maps on E07 at 83.95 cM (proximal marker: CH07_132671839). The QTL with the lower effect (4.5% PVE explained) was located on E10 at 231.46 cM (proximal marker: CH10_94779014).

##### Corolla Colour (*corcol*)

A major QTL (*corcol5.1*), explaining from 40% to 44% of the PVE, was spotted on E05 at 75.48 cM (proximal marker: CH05_37533757) in BT and ML, while *corcol5.2* was located at 39.40 cM (proximal marker CH05_17086140) and explains 19% of PVE in MT. A minor QTL (*corcol10.1*), explaining 7.7%−11.1% of the PVE, was spotted on E10 in BT and ML (not in MT) in the same position at 232.77 cM (proximal marker: CH10_94275882). 

#### 3.3.2. QTL Affecting Speed of Plant Emergence Index

On E02, we mapped the largest effect locus *(sei2.1*), explaining 10.4% of PVE and located at 204.18 cM (proximal marker: CH02_63996392), together with a minor QTL (*sei 2.2*), at 176 cM (proximal marker: CH02_54633733), which explains 8% of the PVE. A further minor QTL was located on E04, at 108.43 cM (proximal marker: CH04_102121728), which explains 10% of PVE. 

### 3.4. Candidate Genes Identification

To find out candidate genes at the identified QTLs in the confidence interval region, we exploited the annotation of the available eggplant genome sequence by searching for genes, including transcription factors, putatively involved in the genetic control of the traits in study. For each QTL, the position, best candidate genes ID, acronym (abbreviation) and predicted function are reported in Table 5.

#### *sei2.1* 

The major QTL associated to *sei* lies on E02 at 204.18 cM and includes four markers in the confidence interval, whose physical positions are quite distant, i.e., 48–54 Mb and 9.2 Mb. The top-linked marker is located at around 54 Mb, in a region containing 15 genes, among which a Pectinesterase 2 and SNL2, a protein involved in response to hormonal stimulus, appeared to be good candidates. The region at 9.2 Mb contains two colocalizing markers: in the interval around 0.3Mb, five genes are present, including two putative NHL6, putatively involved in the response to abscisic acid (ABA).

#### *sei2.2* 

The minor QTL *sei2.2* lies on E02 at 176 cM, in a region of six co-localizing markers located at 60–63 Mb. This region contains 70 genes, some of which may be eligible as candidates, such as the ones associated to the response to abscisic acid (GRDP1 and CAR2), two polyol transporters, a TCP1 and peptide methionine sulfoxide reductase. In the region beneath *sei2.1* and *sei2.2*, a laccase, a zinc finger and a G-box-binding factor 1 putatively involved in seed germination are also present. 

#### *sei4.1* 

In the interval around 0.5 Mb from *sei4.1,* several Serpins-ZX and NRT1/PTR genes protein were identified, as well as an Ent-kaurene oxidase and a B3 domain protein, both involved in gibberellin synthesis. By slightly relaxing the confidence interval (until around 1 M), other genes involved in the signaling pathway of ABA degradation (among which an abscisic acid hydroxylase) were found.

#### *stean10.1,* *lvean10.1,* *flian10.1,* *hyan10.1*

The major QTLs *stean10.1, lvean10.1, flian10.1,* identified at 231.47 cM on E10 in the three environments, as well as the minor QTL *hyan10*.1, lie at around 94.7 Mb, in the top part of the cluster of QTLs associated to anthocyanin amount/coloration intensity and very close to the QTLs for *corcol* and *toan*. In this region, 17 genetic markers are co-segregating, corresponding to a physical interval comprised between 94.54 and 94.88 Mb. The region includes 19 annotated genes, among which a BES1/BRZ1 transcription factor, a DREB2C (dehydration-responsive element-binding protein 2C), an Ankyrin repeat-containing protein and a PPC6-1 (putative protein phosphatase 2C) are eligible as candidates involved in the anthocyanin synthesis. By slightly relaxing the confidence interval, three proteins annotated as regulatory-associated protein of TOR (RAPTOR) are also present.

#### *corcol10.1-toan10.1* 

The QTLs *corcol10.1* and L *toan10.1*, both identified in BT and ML in the cluster on E10 at 232.77cM lean in a region located on E10 containing ten co-localizing markers and physically located at 93.8-94.2 Mb. In this region, the most promising candidate genes are a putative MYB family transcription factor (SMEL_010g352790) and a phosphoinositide phosphatase, SAC8 (SMEL_010g352650), a new class of phosphatase playing a role in vacuolar trafficking. By slightly expanding the region of interest, five genes (SMEL_010g352310, SMEL_010g352320, SMEL_010g352330, SMEL_010g352490 and SMEL_010g352500) were identified and annotated as having predicted 2-oxoglutarate/Fe(II)-dependent dioxygenase activity (ANS). In the same region, three sequences with homology with regulatory-associated protein of TOR (RAPTOR) are localized.

#### *adlan10.1* 

The major QTL *adlan10.1* lies at 236.99 cM on E10, physically located at 94.9–95.08 Mb. A total of seven genes were identified as candidate, five of which were annotated as peroxidases or protein disulfide-isomerases.

#### *stean5.1,* *toan5.2*

The major QTL *stean5.1* identified in the three environments as well as *toan5.2*, specific to BT and ML, underlined a region on E5 in the interval 36.0–36.3 Mb, containing twelve genes. Among them, a class II heat shock protein and a putative BHLH could represent a good candidate. By increasing the confidence interval to the physical position of the marker 3311_PstI_L361, the candidate SMEL_005g236240, annotated as an acetyl-CoA-benzylalcohol acetyltransferase, was found. 

#### *toan5.1-corcol5.1-hyan5.1* 

The major QTL *toan5.1* as well as *corcol5.1* identified in ML and BT and *hyan5.1* in ML lean on E05, in a small region at ~37.5 Mb. Among the seven annotated genes which lie in this interval, two genes annotated as BKI1 (BRI1 kinase inhibitor 1), are eligible as the best candidate. By slightly increasing the region analyzed, two scopoletin glucosyltransferases, as well as a cluster of three genes annotated as encoding cytochrome P450, two zinc fingers and a BHLH-like protein were spotted.

#### *hyan7.1* 

The minor QTL for *hyan7.1* lies on E07 at 83.94 cM, whose confidence interval physical region spanned between 132.76 and 134.02 Mb. More than 80 annotated genes were found, among which the most interesting are a cluster of putative candidates annotated as similar to MYB14−15−58−102, a PPC6−6 and a F-box/kelch-repeat protein. 

#### *stean2.1* 

The minor QTL *tean2.1* lies on E02 at about 30.5 Mbp. A total of five annotated genes were spotted, among which a L10 interacting MYB domain protein, a Heat shock 70 kDa protein and an AKRP—ankyrin repeat domain-containing protein—are eligible as possible candidates.

## 4. Discussion

### 4.1. Genetic Map Construction and Phenotyping 

Studies on eggplant genome organization have received an increasing amount of interest in the last few decades, turning it from a “genomic orphan species” to a crop with a high-quality genomic sequence available [36]. As in several other crops, the low level of polymorphism within the cultivated eggplant germplasm required huge efforts in detecting markers exploitable for linkage mapping purposes [61,62]. 

Several “first generation” inter-specific and intra-specific maps were developed, with the former exploiting a higher genetic polymorphism, but being of minor relevance for marker-assisted breeding [63]. QTLs associated to morphological and plant production traits, as well as parthenocarpy, resistances to fungal and bacterial wilts were identified through bi-parental approaches and genome-wide association (GWA) studies on the basis of the available linkage maps.

An intraspecific F_2_ segregating population, obtained from the same cross from which we developed the RIL population in the present study, proved to be a highly efficient tool for the detection of more than 140 QTLs associated to leaf, flower, plant and fruit traits, fruit biochemical composition and resistances to fungal wilts [22,28,29,30,31]. Our F_7_ RIL population was also previously used for anchoring the “67/3” eggplant genome sequence [36]. Here, we exploited the GBS-derived approach, as applied by Acquadro et al. [64] in eggplant, to develop a new high density linkage map including 7249 SNPs assigned to the 12 chromosomes and spanning 2169.23 cM. Its genetic length is longer than the previously published intra-specific maps [14,22,24,28], as well as the one recently made available by Salgon et al. [34], which spans about 1500 cM and includes 1170 markers.

The newly created map clearly represents a step forward compared to the one we developed for anchoring the genome [36]. This was generated using markers derived from an imputation-based method following low-coverage sequencing of the same mapping population. The pipeline took windows containing 100 SNPs along scaffolds to convert them into genetic markers, which were actually based on the haplotype of 100 SNPs. 

This approach was useful in anchoring the scaffolds to pseudomolecules, but some drawbacks are present, especially for QTL analysis and candidate gene identification. Indeed, this contained three chromosomal locations (E02, E08 and E11), which were split into two different portions. The newly developed map actually includes 12 chromosomes, which in turn may increase the efficiency in identifying candidate regions during QTL analysis. Furthermore, the map developed in this study contains a slightly higher number of gaps shorter than 5cM than the previous one, but some chromosomes have a larger gap. On the other hand, the newly created map is shorter (~500 cM) and contains more markers (1285) (Table S2) than the previous one, resulting in a more dense and saturated map.

Finally, in the map used for anchoring the genome sequence, a marker is based on a window of 100 SNPs, whose size is dependent on the polymorphic level of that specific chromosome regions and whose coordinates in the genome are not well defined. On the contrary, in the new map, each marker is based on a single SNP, allowing us to know the precise position of each marker in the genome and make it possible for a breeder to identify the genes located in a QTL region on the basis the available annotation. Furthermore, GBS data provide information on the SNPs that generated the markers, which is of utility for more targeted analysis.

Our map contains about 24% distorted markers, presumably as a result of the genetic distance between the parents as well as possible preferential or gametic/zygotic selection occurring during the development of our RIL population. However, we included these markers in order to increase the genomic coverage of the genetic map, which reached about 96% of the physical sequence. Indeed, if properly handled, these markers do not cause detrimental effects and increase the potential of QTL mapping, as previously reported [65,66]. The breeding line ‘305E40’, used as the female parent, contains scattered introgressed regions from *S. aethiopicum*, with a large portion on chromosome E02, which includes the locus Rfo-sa1 [29,45]. This may justify the reduced recombination observed not only on E02, but also E09 and E12.

All the anthocyanin-related traits showed a high *h^2^_BS_* (lowest value of 86% for *lvean_BT*) with a high correlation of their phenotypic value among different environments. Similar results were previously reported for some of the traits in the study in the F_2_ population developed from the same cross [28,30]. Transgressive genotypes were infrequent and always deviated towards the less pigmented parent ‘305E40’. The parental line ‘305E40’ produced less vigorous seeds, but about 25% of the RIL population showed a further reduction in the speed of seedling emergence; this is presumably also because we were not able to identify all the QTLs affecting this trait, implying that some other QTLs still remain to be identified. 

Conventionally, a ‘major’ QTL is defined as such when, in addition to justifying a PVE greater than 10% [67], it is conserved in multiple seasons/locations [68,69,70]. We identified at least one major QTL for all the traits in study including *hyan* and *sei*, although in this case the traits were evaluated only in one environment.

### 4.2. QTLs and Underlying Candidate Genes 

The least and the most convincing LOD scores associated with the major QTLs were 3.89 (*sei2.2*) and 37.70 (*flian10.1_MT*), respectively. The explained PVE varied from 10.4% (*sei2.1*) up to 69.3% (*flian10.1_MT*), and most of the identified QTLs were stable across two/three environments, making them potentially useful for marker-assisted selection. On the other hand, some QTLs were identified in just one *(corcol5.2_MT*) or two (like *corcol5.1* or *stean2.1*) environments. This suggests a strong environmental effect on their expression, but also that the rather limited genetic variation in the mapping population did not allow us to fully dissect the genetic bases of these traits [71]. 

#### 4.2.1. Seed Emergency Index

Seed germination is the switch from a dormant embryonic state to a highly active phase of growing. It is also defined as the sum of events that begin with seed imbibition and culminate in the emergence of the embryonic axis (usually the radicle) from the seed coat [72,73]. This progression is controlled by several internal factors, such as auxins, abscisic acid (ABA), cytokinins, ethylene, and GA content and balance, as well as environmental factors that include water availability, temperature, and light [74]. Eggplant, as with most of the *Solanum* species, is mainly propagated by seeds, whose vigor influences their germination and seedling emergence performance. Seed dormancy, low uniformity and poor germination rate have been documented in many eggplant accessions as well as in wild and allied species, including those employed as rootstock or those useful for introgression breeding [11,75,76,77,78,79]. 

Here, we reported, for the first time, three QTLs associated to seed vigor in eggplant, assessed by evaluating the speed of emergence index. Only one major QTL was spotted, suggesting that other key regions controlling this trait are still to be identified. 

However, interestingly, both a major and minor QTL (i.e., *sei2.1* and *sei2.2*) were detected on chromosome E02, with *sei2.1* inherited from the female parent ‘305E40’. As previously pointed out, this breeding line harbors on chromosome E02 an introgressed fragment from *S. aethiopicum*, [28,36], associated to the *Fusarium oxysporum* resistance locus *Rfo-sa1* [45]. Thus, we could speculate that the introgressed portion of *S. aethiopicum* genome might be also involved in the genetic control of this trait, as this allied species usually displays a delayed germination with respect to eggplant. 

The introgressed region may also be responsible of an inaccurate positioning of the genomic sequences in this region, and, consequently, in a reduction in the QTL mapping efficiency. Indeed, the candidate genes encompassing *sei2.1* are physically located both at 48–54 Mb and 9.2 Mb on E02. In the first large region, a paired amphipathic helix protein Sin3-like 2 could be a good candidate gene as it belongs to a class of proteins, involved in the response to hormonal stimuli and in the seed dormancy breakdown [80], while the NHL6 genes identified at 9 Mb have been reported to play an important role in the abiotic stress-induced abscisic acid (ABA) signaling and biosynthesis, acting as positive regulator of ABA-mediated seed germination inhibition [81]. 

*Sei2.2* overlies some candidates annotated as similar to previously described genes involved in the ABA response and seed dormancy breakdown: two membrane C2-domain abscisic acid-related proteins (CAR2) [82] and a GRDP1—glycine-rich domain-containing protein 1 [83]. Furthermore, two TCP1 encoding genes and a peptide methionine sulfoxide reductase should also be considered as similar genes are involved in the repair mechanism during seed dormancy release in *Arabidopsis* and the increase in *M. truncatula* seed longevity by reducing the protein oxidation damage [84,85], respectively. 

Several Serpins-ZX and NRT1/ PTR coding-genes were identified in the *sei4.1* region. The Serpin gene family has gained attention in wheat and barley for its role in grain development, but these genes could play a possible role in the mobilization of sugars during germination by enhancing β-amylase enzymatic activity and preventing β-amylase aggregation during oxidative stress [86]. The cluster of NPF6/NRT1–1 nitrate or di/tripeptide transporters, also spotted in *sei4.1*, are potentially involved in nitrate sensing and signaling [87,88], and genes belonging to this class are reported to regulate seed development, germination and dormancy cycling in fava bean and *Arabidopsis* [89,90,91], with a possible involvement in the ABA transport [92]. Nitrate itself is reported as a signal molecule that controls several aspects of plant development including seed dormancy, as higher nitrate accumulation in mother plants leads to lower seed dormancy [93].

Good candidates for future studies might also be other genes in the same region encoding an Ent-kaurene oxidase and two B3 domain proteins, all involved in GA biosynthesis: the latter are regulating factors of the pathway in which the former is a key enzyme, and it is reported that suppressive mutations in the coding region of both genes cause a delay in seed germination and seedling development [94,95].

#### 4.2.2. Anthocyanins

Anthocyanins are among the most represented flavonoid compounds in plants and are responsible for the pigmentation of many flowers and fruits. They have an essential eco-physiological role in attracting pollinators and seed dispersers [96,97] and are also implicated in the response against biotic and abiotic stresses [98,99]

The genetic control of anthocyanin formation, distribution and accumulation has been widely studied in Solanaceae species [38,39,40,41,42,43,100,101]. This was long thought to be a complex trait in eggplant, involving several loci with assumed epistatic interactions and/or pleiotropic effects [102,103]. More recently, QTL-related studies allowed us to identify the chromosome regions involved in anthocyanin distribution in eggplant tissues and organs, highlighting their synteny with tomato [28,30,32,44] and, thanks to the recent availability of an high quality eggplant genome sequence coupled with metabolomic analyses [37], allowed us to localize putative candidate genes.

As previously observed [28,30,32], our ultra-high density genetic linkage map confirmed that the clusters on E10 and E05 are involved in the pigmentation of eggplant tissues, which may be associated with two different aspects of the anthocyanin synthesis among tissues, but likely control different processes linked to anthocyanin accumulation in diverse tissues. Indeed, the cluster on E10 is mainly prominent for anthocyanin production and accumulation in the vegetative plant organs, except in the corolla of the flower, whose pigmentation is governed by the major QTL on E5 with a smaller contribution by a minor QTL on E10. Conversely, the cluster on E05 contains QTLs more likely associated with the anthocyanin tonality (in flower, with *frucol*, and in general in all the vegetative tissues, represented by *toan*) and with the accumulation of anthocyanins in hypocotyl (*hyan).* Although the phenotypic data available for *hyan* were only collected in one environment, the combined results with *stean* QTLs seem to suggest that both 5 and 10 are involved, but with stronger specific effect on anthocyanin accumulation of *hyan5.1* in the hypocotyl at plantlet stage and of *stean10.1* in the stem of the fully developed plant. Overall, the joint effect of both E05 and E10 QTLs could impact on *hyan*, *stean*, *corcol* and *toan* through an interaction between genes, influencing both tonality and anthocyanin intensity.

#### 4.2.3. Anthocyanin Related Candidate Genes Identifications

The biosynthesis of anthocyanin is one of the most studied pathways in plants, with most of the genes encoding for enzymes and regulatory transcription factors (TFs) identified in several plant species, including Solanaceae [3,104]. The anthocyanin pathway is under the control of many early (EBGs) or late (LBGs) biosynthetic genes, with the former involved in the first steps of biosynthesis of flavonols and other flavonoid compounds and the latter involved in the ensuing steps of the pathway until the final steps of decoration, leading to different anthocyanin compounds. Each enzymatic step of this complex pathway is finely tuned by co-activators independent and functionally redundant R2R3-MYB regulatory proteins which regulate the expression of structural genes, alone or in complexes with other TFs belonging to the basic helix-loop-helix (BHLH) family. The control of the biosynthetic pathway is strongly dependent on tissue, developmental stage and environment, and has only been partially elucidated in eggplant with regard solely to the fruit peel coloration [36,103,105,106,107,108].

##### Cluster on Chromosome E10

The cluster identified on E10 lies in a region of 1.5 Mb, between 93.5 and 95 Mb, containing three clusters of colocalizing QTLs. 

We spotted, next to the upper limit of the cluster and close to *toan*, five genes predicted as ANS, which might be involved in the oxidation of leucocyanidin, in the second to last step of anthocyanin biosynthesis [109]. A further characterization highlighted that these genes are located in a genomic region of the parental line ‘67–3’ containing retrotransposon-like sequences, which could alter the expression pattern of nearby genes [110]. Within the QTL for *toan* at 232.77 cM, we identified a putative MYB transcription factor and a phosphoinositide phosphatase, belonging to a class of phosphatases that plays a key role in abiotic stress response, vacuolar trafficking and anthocyanin accumulation [111]. 

Lying within *stean10.1*, we identified an ankyrin repeat-containing protein coding gene together with a dehydration-responsive element-binding protein 2C and a PPC6–1 (protein phosphatase 2C). Ankyrin repeat proteins were reported to be involved in the anthocyanin synthesis pathway [112], while the other two candidates are putatively involved in the response to abiotic stresses and detoxification [113]. Finally, alongside *adlan10.1,* we identified five peroxidases coding genes which may be involved in the degradation of anthocyanin, influencing the overall coloration of the tissues where they are expressed. Indeed, enzymatic degradation has been considered to be responsible for anthocyanin breakdown in plants, leading to pigment concentration reduction and colour fading [114]. Recent studies have shown that PODs and laccases (LACs) are responsible for anthocyanin catalysis [115,116], and also, in combination with some environmental factors, such as high temperature and low light density, were reported to enhance the peroxidase activity [104,117].

##### QTL cluster on Chromosome E05

The region identified on E05 which controls *stean*, *toan*, *hyan* and *corcol* contains two slightly separate clusters. The upper region, including the QTLs for *toan5.2* and *stean5.1*, was already spotted by Barchi et al. [28] as a genomic region involved in the control of several anthocyanin-related traits (such as *stean*) and the corolla color. In the same position, Toppino et al. [30] mapped QTLs associated to peel fruit color as well as to the presence and amount (determined by HPLC) of D3R and nasunin, the two different anthocyanins in the eggplant peel. Analogous QTLs in the distal portion of E05 were also previously identified by GWAS approaches [32,44]. 

Our candidate gene search highlighted the presence of an acetyl-CoA-benzylalcohol acetyltransferase (AAT), for which we speculate a function in the aromatic group decoration as the last step of the anthocyanin biosynthetic pathway. Furthermore, the distribution and dominance relationships strongly support the hypothesis that AAT is active in ‘67/3’ and inactive in ‘305E40’, and thus responsible of the acetylation of the D3R glucosidic group [118] and the subsequent conversion into nasunin. The comparison of the Illumina sequencing data available for the two parental lines [36] revealed a 1bp indel which could determine a loss-of-function mutation in the *305E40_AAT* CDS sequence, opening the path to a deeper functional study of this gene. 

The cluster of QTLs for *corcol, hyan* and *toan* on E5 is proximal to *toan5.1* and *stean*; in this region, two scopoletin glucosyltransferase coding genes, involved in the phenylpropanoid pathways [119], were spotted, alongside four genes annotated as cytochrome P450, known as playing pivotal roles in the biosynthesis of plant secondary metabolites, including phenylpropanoids and phytoalexins [120,121]. 

##### QTLs for *hyan7.1* and *stean2.1*

In the *hyan7.1* region, a cluster of MYB genes were identified, with homology to sequences known to be involved in the phenylpropanoid pathway and more specifically in the stilbene biosynthesis in *Vitis* [122], lignin in *Arabidopsis* [123] and anthocyanin in forage legumes [124]. In the same region, other interesting candidate genes are a PPC6−6 (probable protein phosphatase 2C) and a F-box/kelch-repeat protein, a class of regulators reported to be associated to phenylpropanoid pathway [125]. 

Finally, for *stean2.1,* a valid candidate gene was an AKRP—ankyrin repeat domain-containing protein—which was reported to be involved in the anthocyanin synthesis pathway [112]. 

## 5. Conclusions

Our results demonstrate that the newly developed map, supported by genome annotation, supplies a key tool to gather valuable information for QTL fine mapping, candidate gene identification, and for the development of molecular markers suitable for identifying favorable alleles, and thus increasing the precision and efficiency of selection in breeding. Our high-density intraspecific map made it possible not only to validate previously reported QTLs, but also to identify new ones associated with the plant anthocyanin pigmentation intensity and tonality, as well as to better define their underlying chromosomal regions. Thanks to the availability of genome annotation, it was also possible to provide a set of relevant candidate genes involved in the anthocyanin biosynthetic process and regulation, some of which are already the subject of ongoing studies.

Finally, the map allowed us to identify the first QTLs affecting seed vigor in eggplant, as measured by the speed of seedling emergence from soil. The identification of the genetic bases of this trait are of key importance, since seed germination and seedling emergence represent two of the most vulnerable phases of a crop cultivation cycle, and less vigorous seeds, other than reducing the crop competitiveness toward weeds, increase the exposure of seedlings to abiotic (drought, heat) and biotic (soil-borne pests) stresses. On the whole, the QTLs we detected provide important knowledge on the genomic region linked physiological and phenotypic properties in eggplant which may be usefully exploited in future breeding programs.

## Figures and Tables

**Table 1:** List of the traits analyzed and their code, means, standard deviations (SD), coefficients of variation (cv), broad sense heritability and transgressive genotypes for the traits in study.

Trait	Trait Code	305E40 Mean	±SD	67/3 Mean	±SD	F1 Mean	±SD	cv	RIL pop Means	±SD	cv	Shapiro-Wilks	Skewness	SE	Kurtosis	SE	Heritability	Transgressive Respect 305E40	Transgressive Respect 67/3
Adaxial Leaf Lamina Anthocyanin	*adlan_BT*	0.42	0.20	5.00	0.00	1.67	1.17	0.70	1.76	1.58	0.89	0.87	0.72	0.08	−0.75	0.16	0.97	2	0
	*adlan_ML*	0.42	0.20	4.67	0.41	2.25	0.42	0.19	2.04	1.58	0.77	0.90	0.43	0.08	−1.15	0.16	0.94	0	0
	*adlan_MT*	0.00	0.00	2.67	0.87	1.11	0.60	0.54	0.69	1.03	1.49	0.71	1.63	0.07	2.14	0.13	0.92	0	0
Stem Anthocyanin	*stean_BT*	2.17	0.25	5.00	0.00	4.83	0.25	0.05	3.80	1.02	0.27	0.92	−0.62	0.07	−0.14	0.13	0.96	3	0
	*stean_ML*	2.33	0.43	5.00	0.00	4.78	0.36	0.08	3.80	0.98	0.26	0.92	−0.35	0.07	−0.80	0.13	0.96	0	0
	*stean_MT*	1.11	0.33	4.89	0.33	4.44	0.53	0.12	3.39	1.08	0.32	0.91	−0.22	0.07	−0.53	0.13	0.95	0	0
Leaf Venation Anthocyanin	*lvean_BT*	1.67	0.26	5.00	0.00	4.33	0.26	0.06	3.61	1.33	0.37	0.76	4.26	0.08	71.86	0.16	0.86	1	0
	*lvean_ML*	2.75	0.42	4.92	0.20	4.75	0.27	0.06	3.74	0.99	0.27	0.92	−0.54	0.08	−0.24	0.16	0.87	2	0
	*lvean_MT*	1.00	0.71	4.22	0.97	4.44	1.01	0.23	3.06	1.53	0.50	0.91	−0.38	0.07	−0.95	0.13	0.87	0	0
Flower Anthocyanin Intensity	*flian_BT*	2.28	1.12	5.00	0.00	4.89	0.22	0.05	4.27	0.89	0.21	0.79	−1.05	0.07	0.40	0.13	0.94	0	0
	*flian_ML*	2.89	0.89	4.89	0.33	4.67	0.43	0.09	3.95	1.06	0.27	0.83	−0.75	0.07	−0.06	0.13	0.95	0	0
	*flian_MT*	3.22	1.48	4.89	0.33	5.00	0.00	0.00	4.12	0.98	0.24	0.84	−0.79	0.07	−0.43	0.13	0.95	0	0
Anthocianin Tonality	*toan_BT*	1.00	0.00	5.00	0.00	3.00	0.00	0.00	3.02	1.86	0.62	0.72	−0.02	0.11	−1.85	0.23	0.94	0	0
	*toan_ML*	1.00	0.00	5.00	0.00	3.00	0.00	0.00	3.21	1.86	0.58	0.71	−0.21	0.11	−1.80	0.22	0.89	0	0
Corolla Colour	*corcol_BT*	1.00	0.00	5.00	0.00	4.11	0.33	0.08	3.00	1.78	0.59	0.76	−0.01	0.07	−1.78	0.13	0.97	0	0
	*corcol_ML*	1.00	0.00	5.00	0.00	3.33	1.32	0.40	3.23	1.78	0.55	0.76	−0.20	0.07	−1.75	0.13	0.97	0	0
	*corcol_MT*	4.33	1.00	5.00	0.00	4.67	0.50	0.11	4.49	1.10	0.24	0.51	−2.08	0.07	3.26	0.13	0.87	0	0
Hypocotyl Anthocyanin	*hyan*	0.67	0.29	4.83	1.26	3.00	0.50	0.17	2.88	1.34	0.47	0.94	−0.18	0.12	−0.98	0.23	0.98	1	0
Speed of emergence index	*sei*	11.67	1.53	5.67	1.53	5.67	1.15	0.20	13.65	3.00	0.22	0.90	1.27	0.12	4.80	0.23	0.98	44	0

**Table 2:** Parameters associated with the framework eggplant genetic map. ^a,b^ The overall physical start and end positions of all markers of each linkage group. ^c^ The distance between the linkage group (LG) physical start and LG physical end indicates the overall physical span of all the markers of each linkage group in a particular chromosome.

												Marker Density		Ratio cM/Mb (Estimated Recombination Rate)
LG/Chromosome	Size (cM)	Size (Mp)	Markers	LG Physical Start (bp)^a^	LG Physical End (bp)^b^	Physical Span (Mb)^c^	Max Gap (cM)	Gap <5 cM	Markers from Different CH	Markers from CH0	Distorted Markers	cM	kb	
E01	180.09	136.53	426	1,761,388	136,521,595	134.76	14.89	0.984	0	0	54	0.4	321.3	1.32
E02	326.00	83.34	1454	5796	77,157,371	77.15	6.71	0.999	9	3	1425	0.2	57.4	3.91
E03	148.51	97.01	408	36,214	82,766,928	82.73	18.00	0.985	9	19	7	0.4	238.4	1.53
E04	119.40	105.67	258	1,557,104	105,339,521	103.78	14.46	0.973	0	0	0	0.5	411.2	1.13
E05	157.15	43.85	211	2,017,627	43,756,820	41.74	13.75	0.957	0	13	72	0.7	208.8	3.58
E06	126.94	108.97	838	2187	99,842,165	99.84	12.19	0.995	24	104	8	0.2	130.2	1.16
E07	115.69	142.38	535	16,820	140,599,277	140.58	14.79	0.994	1	16	36	0.2	266.6	0.81
E08	183.94	109.58	730	2123	10,680,425	106.80	8.89	0.999	0	2	1	0.3	150.3	1.68
E09	107.06	36.10	230	11,386	34,244,824	34.23	18.18	0.978	1	15	1	0.5	157.6	2.97
E10	281.23	106.64	386	701,005	106,482,885	105.78	22.29	0.956	0	0	68	0.7	277.0	2.64
E11	141.34	72.29	231	213,621	72,208,518	71.99	12.24	0.974	3	59	39	0.6	314.3	1.96
E12	281.90	100.42	1542	10,639	96,335,130	96.32	22.45	0.995	0	1	33	0.2	65.2	2.81
Total	2169.23	1142.80	7249.00			1095.72			47	232	1744	0.4	216.5	2.12

**Table 3:** Inter-trait Spearman correlations assessed in the mapping population. In green, significant correlation at *p* < 0.05, in blue at *p* < 0.01.

	*adlanML*	*adlanMT*	*corcolBT*	*corcolML*	*corcolMT*	*flianBT*	*flianML*	*flianMT*	*sei*	*hyan*	*lveanBT*	*lveanML*	*lveanMT*	*steanBT*	*steanML*	*steanMT*	*toanBT*	*toanML*
*adlanBT*	0.90	0.81	0.21	0.21	0.21	0.43	0.45	0.50	0.02	0.19	0.49	0.42	0.66	0.38	0.38	0.39	0.20	0.16
*adlanML*		0.83	0.20	0.19	0.18	0.37	0.43	0.45	0.00	0.17	0.40	0.41	0.66	0.32	0.37	0.38	0.19	0.18
*adlanMT*			0.15	0.15	0.18	0.39	0.42	0.45	0.00	0.11	0.42	0.39	0.65	0.30	0.37	0.36	0.12	0.15
*corcolBT*				0.85	0.60	0.57	0.53	0.53	0.03	0.69	0.57	0.61	0.42	0.74	0.65	0.69	0.92	0.83
*corcolML*					0.68	0.51	0.54	0.54	0.05	0.68	0.58	0.58	0.44	0.75	0.68	0.71	0.81	0.91
*corcolMT*						0.42	0.37	0.41	−0.12	0.48	0.45	0.44	0.34	0.60	0.56	0.55	0.58	0.63
*flianBT*							0.85	0.81	0.04	0.42	0.80	0.79	0.55	0.79	0.77	0.73	0.50	0.45
*flianML*								0.85	0.02	0.37	0.83	0.81	0.60	0.75	0.83	0.78	0.42	0.47
*flianMT*									0.02	0.44	0.82	0.78	0.61	0.77	0.80	0.80	0.45	0.46
*sei*										0.09	0.07	−0.01	−0.02	0.05	−0.01	0.00	0.07	−0.01
*hyan*											0.52	0.43	0.27	0.65	0.53	0.54	0.66	0.65
*lveanBT*												0.83	0.59	0.83	0.82	0.80	0.52	0.50
*lveanML*													0.52	0.78	0.83	0.83	0.55	0.57
*lveanMT*														0.54	0.59	0.62	0.35	0.36
*steanBT*															0.88	0.88	0.70	0.68
*steanML*																0.91	0.59	0.62
*steanMT*																	0.62	0.65
*toanBT*																		0.84

**Table 4:** QTL detected in the mapping population. For each trait the chromosomal location (Chr.), the genome-wide thresholds (GW) at *p* = 0.05 (as determined from 1000 permutations) are indicated.

	Battipaglia									Montanaso Lombardo									Monsampolo del Tronto								
Trait	GW	QTL	Position			LOD	CI	PVE	A	GW	QTL	Position			LOD	CI	PVE	A	GW	QTL	Position			LOD	CI	PVE	A
			Chr.	cM	Locus							Chr.	cM	Locus							Chr.	cM	Locus				
*adlan*	*3.3*	adlan10.1_BT	E10	236.98	CH10_95003635	7.59	236.98	21.0	−0.6002	3.3	adlan10.1_ML	E10	236.98	CH10_95003635	7.47	236.98	20.4	−0.5872	3.3	adlan10.1_MT	E10	236.98	CH10_95003635	6.21	236.987	17.7	−0.3465
*stean*	*3.2*									3.2	stean2.1_ML	E02	88.73	CH02_30555633	4.90	88.73	5.6	−0.2294	3.2	stean2.1_ML	E02	88.73	CH02_30555713	7.71	88.733	9.5	−0.3255
		stean5.1_BT	E05	66.39	CH05_36124744	15.58	62.45–66.51	21.5	−0.4263		stean5.1_ML	E05	66.39	CH05_36124744	9.22	66.39	13.0	−0.3156		stean5.1_ML	E05	64.51	CH05_36124744	9.70	62.45–64.516	16.8	−0.3882
		stean10.1_BT	E10	231.46	CH10_94779014	26.93	231.46	45.1	−0.6000		stean10.1_ML	E10	231.46	CH10_94779014	25.92	231.46	48.1	−0.5894		stean10.1_MT	E10	231.46	CH10_94779014	20.76	231.465	40.3	−0.5851
*lvean*	*3.2*	lvean10.1_BT	E10	231.46	CH10_94779014	32.78	231.46	64.2	−0.7883	3.2	lvean10.1_ML	E10	231.46	CH10_94779014	24.92	231.46	53.2	−0.6202	3.2	lvean10.1_MT	E10	231.46	CH10_94779014	10.52	231.465	28.2	−0.5629
*flian*	*3.1*	flian10.1_BT	E10	231.46	CH10_94779014	29.26	231.46	59.5	−0.5680	3.1	flian10.1_ML	E10	231.46	CH10_94779014	31.64	231.46	61.9	−0.6544	3.1	flian10.1_MT	E10	231.46	CH10_94779014	37.70	231.465	69.3	−0.7393
*toan*	*3.4*	toan5.1_BT	E05	75.48	CH05_37533757	17.89	75.48	35.7	−110.9	3.4	toan5.1_ML	E05	75.48	CH05_37533757	22.92	75.48	43.9	−116208									
		toan5.2_BT	E05	66.39	CH05_36076199	3.72	61.55–66.39	5.2	−0.6245		toan5.2_ML	E05	66.39	CH05_36124744	7.44	61.55–66.39	8.7	−0.7422									
		toan10.1_BT	E10	232.77	CH10_94275882	5.67	232.77	9.3	−0.5498		toan10.1_ML	E10	232.77	CH10_94275882	4.82	232.77	6.8	−0.4464									
*corcol*	*3.3*	corcol5.1_BT	E05	75.48	CH05_37533757	22.39	75.484	40.2	−107.477	3.3	corcol5.1_ML	E05	75.48	CH05_37533757	25.31	75.48	45.2	−112175	3.3	corcol5.1_MT	E05	39.39	CH05_17086140	6.88	39.397	19.3	−0.3440
		corcol10.1_BT	E10	232.77	CH10_94275882	7.88	232.77	11.1	−0.5493		corcol10.1_ML	E10	232.77	CH10_94281914	5.95	232.77	7.7	−0.4507									
*hyan*										3.3	hyan5.1	E05	75.48	CH05_37533757	23.72	75.48	48.1	−0.9234									
											hyan7.1	E07	83.94	CH07_132761839	3.93	79.32–83.94	5.8	−0.3223									
											hyan10.1	E10	231.46	CH10_94684020	3.37	230.12–233.45	4.5	−0.2807									
*sei*										3.0	sei2.1	E02	204.18	CH02_54633733	5.23	204.18	10.4	−5.689									
											sei2.2	E02	176.24	CH02_63996392	3.89	176.24	7.6	5.109									
											sei4.1	E04	108.42	CH04_102121728	4.81	102.22–114.85	9.7	−0.8733									

**Table 5:** Candidate genes spotted within the interval of detected QTLs. For each QTL, position, best candidate genes ID, acronym (abbreviation) and putative function are provided.

QTL	Approximative Position	Gene		Predicted Function
		IDs SMEL_	Abbreviation	
*sei 2.1*	9.2 Mb	002g153950.960	NHL6	2x NDR1/HIN1-like protein 6
	54 Mb	002g158620	PECS−2.1	Pectinesterases 2
		002g158940	SNL2	Paired amphipathic helix protein Sin3-like 2
*between sei 2.1 and sei 2.2*	~54–60 Mb	002g159100	LAC11	Laccase
		002g159470	ENY	Zinc finger ENHYDROUS
		002g159370.380	PLT6	2x polyol transporter
		002g159480	GBF1	G-box-binding factor 1
*sei 2.2*	60–63 Mb	002g160070.080	CAR2	2x C2-Domain Abscisic Acid-Related Proteins
		002g160170	GRDP1	Glycine-rich domain-containing protein 1
		002g159720	TCP1	T-complex protein 1 subunit zeta 1
		002g159870	MSR4	Peptide methionine sulfoxide reductase
*sei 4.1*	102 Mb	004g219910.920		2x Serpins-ZX
		004g220200–220	NPF4.5/NPF4.3	3x NRT1/ PTR protein fam 4.5/4.3
		004g220270	KO	Ent-kaurene oxidase
		004g220280	REM16	B3 domain transcription factor
		004g220780	GAF1	Zinc finger GAI-ASSOCIATED FACTOR 1
	~102 Mb	004g221390		abscisic acid 8’ hydrolase 4
		004g221470	BZIP44	bZIP transcription factor 44
*toan10.1 corcol 10.1*	~94 Mb	010g352310–490	ANS	4x 2-oxoglutarate/Fe(II)-dependent dioxygenase
		010g352500	JRG21	2-oxoglutarate/Fe(II)-dependent dioxygenase
	94 Mb	010g352790		Myb family transcription factor
		010g352650	SAC8	Phosphoinositide phosphatase
*lvean 10.1 stean 10.1 flian10.1 hyan 10.1*	94.7 Mb	010g352910	BES1/BZR1	BES1/BZR1 transcription factor
		010g352980		Ankyrin repeat-containing protein
		010g352930	DREB2C	Dehydration-Responsive Element-Binding Protein 2C
		010g353040	PPC6–1	protein phosphatase 2C
	~94.7 Mb	010g353090–110	RAPTOR	3x RAPTOR - Regulatory-associated protein of TOR
*adlan 10.1*	95 Mb	010g353170–200		5x peroxidase
		010g353200		Protein disulfide-isomerase
*hyan 7.1*	132.7 Mb	007g289310–410	MYBs	6x similar to MYB15/14/58/102
		007g289700.710	NDB	2x NAD(P)H dehydrogenase
		007g289780		F-box/kelch-repeat protein
		007g289410	PPC6–6	protein phosphatase 2C
*stean 2.1*	30.5 Mb	002g155860		Ankyrin repeat-containing protein
		002g155880	LIMYB	L10-interacting MYB domain-containing protein
		002g155890	HSP70	Similar to Heat shock 70 kDa protein
		002g155950.970	LARP1C	2x La-related protein 1C-like
*stean 5.1 toan 5.2*	36.2 Mb	005g235930	HSP	17.3 kDa class II heat shock protein
		005g236210	BHLH93	similar to Transcription factor bHLH93
	~36.2 Mb	005g236240	AAT	Acetyl-CoA-benzylalcohol acetyltransferase
*hyan 5.1 toan 5.1 corcol 5.1*	~37.5 Mb	005g236840–90		7x Calmodulin-like genes
	37.5 Mb	005g236910.20	BKI1	2x BR1 kinase inhibitors
	~37.5 Mb	005g236720.30	TOGT1	2x Scopoletin glucosyltransferase
		005g236480	BHLH84	Transcription factor bHLH84
		005g236490.00	AZF3,ZAT10	2x Zinc finger protein
		005g236570–620	CYP81Q32,VQ31	cytochrome p450

## Supplementary Materials

The following are available online at https://www.mdpi.com/2073-4425/11/7/745/s1, Figure S1: Distribution of sequenced reads, after quality cleaning and trimming procedures, across the parental lines and F_1_ (first three samples) as well as the RIL mapping population (in million reads); Figure S2: Eggplant linkage map. Marker names are shown to the right of each chromosome, with map distances (in cM) shown on the left; Figure S3: Heat map representing pairwise recombination fractions with LOD scores for each marker on all 12 chromosomes; Figure S4: Marey Map plots of SNPs mapped to positions on the 12 *S. melongena* chromosomes vs. their physical positions on the v3. eggplant pseudomolecules from Barchi et al. [36]; Table S1: Markers name, chromosome, genetic position of all markers on the map and RILS graphical haplotypes are reported. The color ‘orange’ represents homozygous alleles from the “305E40” parent, ‘blue’ represent homozygous alleles from “67/3” parent, ‘green’ as heterozygous alleles and white as missing data; Table S2: Parameters associated with the eggplant genetic map compared to the previous developed by Barchi et al. [36].
