# Supplementary figures and images for: A New Intra-Specific and High-Resolution Genetic Map of Eggplant Based on a RIL Population, and Location of QTLs Related to Plant Anthocyanin Pigmentation and Seed Vigour
Source: Genes (Basel). 2020 Jul 4;11(7):745. doi: 10.3390/genes11070745 (PMC7397344; doi:10.3390/genes11070745)

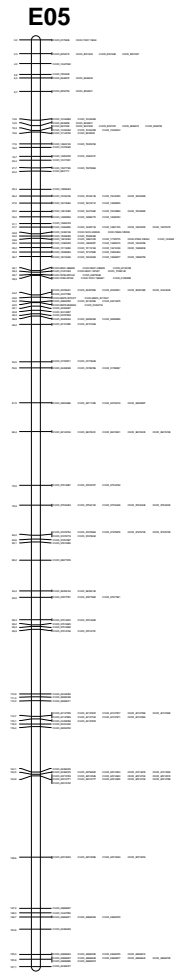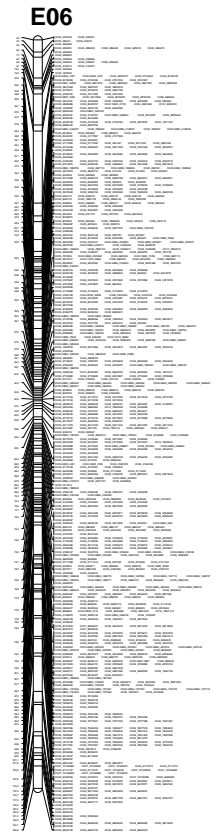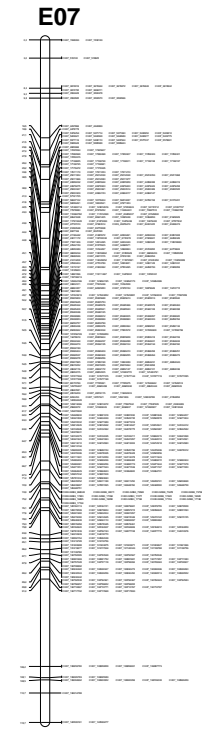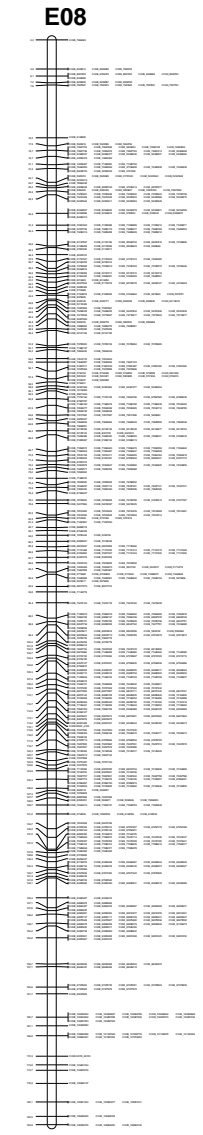

Supplement: Supplementary file 1 [file genes-11-00745-s001.zip › Supplementary_Figures_Tables/Figure_S2.pdf]

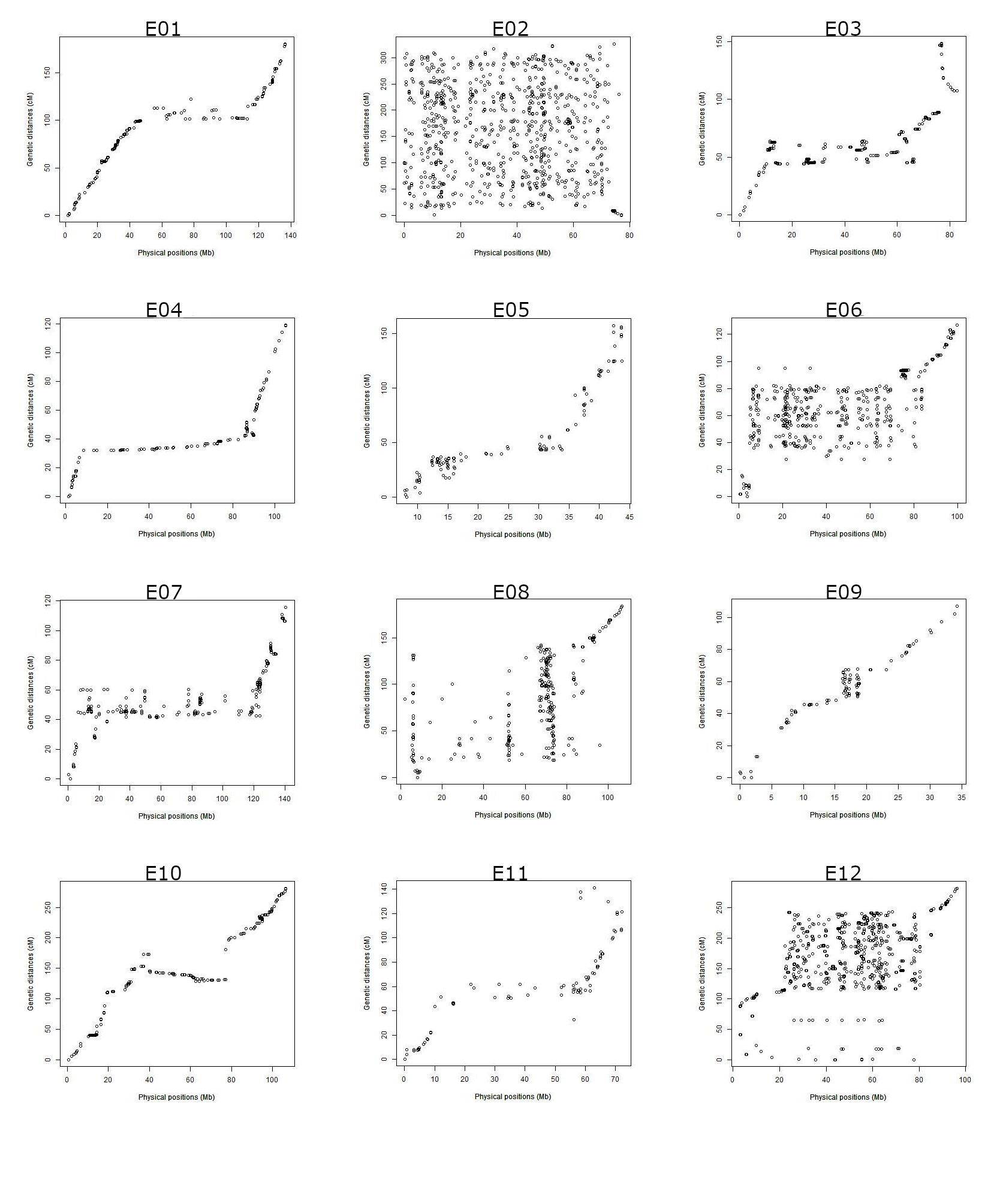

Supplement: Supplementary file 1 [file genes-11-00745-s001.zip › Supplementary_Figures_Tables/Figure_S4.png]
